# Low Frequency Stimulation Reverses the Kindling-Induced Impairment of Learning and Memory in the Rat Passive-avoidance Test

**DOI:** 10.29252/NIRP.BCN.9.1.51

**Published:** 2018

**Authors:** Khadijeh Esmaeilpour, Vahid Sheibani, Mohammad Shabani, Javad Mirnajafi-Zadeh, Zeinab Akbarnejad

**Affiliations:** 1. Neuroscience Research Center, Institute of Neuropharmacology, Kerman University of Medical Sciences, Kerman, Iran.; 2. Department of Physiology, Faculty of Medical Sciences, Tarbiat Modares University, Tehran, Iran.

**Keywords:** Epilepsy, Learning and memory, Low-frequency stimulation, Kindling

## Abstract

**Introduction:**

The life quality of patients with epileptic seizures is highly affected by cognitive deficits. Low Frequency Stimulation (LFS) is a novel approach for the treatment of pharmacoresistant epilepsy. The main goal of this research is investigating the possible effect of LFS on seizure-induced cognitive dysfunction.

**Methods:**

To this end, the kindled animal were prepared via CA1 electrical stimulation in a semi-rapid way (12 stimulations/day). A group of animals were stimulated with LFS, 4 times at 30 s, 6 h, 24 h, and 30 h after the last kindling stimulation. Applied LFS was administered in 4 packages every 5 minutes. The packages were designed with 200 monophasic 200 monophasic square wave pulses of 0.1 ms duration at 1 Hz. The passive-avoidance test was conducted on all animals in order to measure the learning and memory behavior.

**Results:**

Hippocampal kindled rats showed deficits in learning and memory when passive avoidance test was performed. Application of LFS reversed the impairment in learning and memory behavior in kindled rats. At the same time, LFS markedly diminished kindling-induced neuronal loss and atrophy in the hippocampus.

**Conclusion:**

LFS may have some protection against seizure-induced cognitive damage in kindled rats.

## 1. Introduction

Attention deficits, as well as learning and memory impairment count as cognitive impairments. These impairments are prevalent in epileptic patient (Holmes 1991; Mula & Trimble, 2009). Numerous studies in animal models have shown that kindling can increase hippocampal-dependent memory impairments, such as spatial memory in Morris Water Maze (MWM) (Beldhuis, Everts, Van der Zee, Luiten, & Bohus, 1992; Gilbert, McNamaira, Corcoran, 1996; Cammisuli et al., 1997; Sutherland, Leung, Weisend, Schlife, & McDonald, 1997), working memory in Radial maze (Leung, Boon, Kaibara, & Innis, 1990), and emotional memory in shuttle box (Grecksch, Becker Gadau, & Matthies, 1991; Genkova-Papazova, 1995; Pohle, Becker, Grecksch, Juhre, & Willenberg, 1997; Hannesson, Howland, Pollock, Wallace, & Corcoran, 2001; Szyndler et al., 2002). As a new emerging approach, deep brain stimulation is used for the treatment of pharmacoresistant epilepsy (Durand & Bikson, 2001; Li & Mogul, 2007; Fisher et al., 2010; Cukiert, Cukiert, Burattini, & Lima, 2014). Former researchers have proved that LFS would induce a suppressive effect on epilepsy (Yamamoto et al., 2002; Goodman, Berger, Tcheng, 2005) and kindling-induced potentiation (Mohammad-Zadeh et al., 2007).

We have shown that LFS application in kindled animals can improve impairments in spatial learning and memory, which is due to hippocampal kindling in both MWM (Esmaeilpour, Sheibani, Shabani, & Mirnajafi-Zadeh, 2017) and Y- maze task (Ghafouri et al., 2016) paradigms. As one of the most frequent seizure disorders, temporal lobe epilepsy is associated with lesions in limbic structures, such as the amygdala and hippocampus (Elger, Helmstaedter, Kurthen, 2004; Cristinzio Perrin & Vuilleumier, 2007). Emotional learning and memory are dependent on the operation of neuronal circuits within the medial region of the temporal lobe, essentially hippocampus and amygdale (Cahill & McGaugh, 1998).

According to the literature, seizure can impair emotional learning and memory in passive and active avoidance paradigms in animal (Grecksch et al., 1991; Genkova-Papazova, 1995; Pohle et al., 1997; Hannesson et al., 2001; Szyndler et al., 2002) and also emotional experience and behavior in human (Cendes et al., 1994; Lanteaume et al., 2006). While previous studies have supported the improving effect of LFS on spatial learning and memory impairment (induced by kindling), a question is raised owning to the connections between hippocampus and amygdale. We investigated whether LFS application in kindled animals will lead to improvement effect in kindling-induced impairment in emotional learning and memory in passive avoidance task.

## 2. Methods

### 2.1. Animals

The study rats were Wistar rats with 200–250 g in weight. The animals were kept inside cages in group of 4 and put under a light-dark cycle at 23^°^C±1^°^C. All the animals were given food and water ad libitum. Regional Ethics Committee of KNRC/93–53 approved the study and the Guide for the care and use of laboratory animals was followed.

### 2.2. Surgery and kindling procedure

The rats were placed in a stereotaxic frame after being anesthetized with ketamine and xylazine (100 and 10 mg/kg, respectively, IP). The scalp was cut, a hole was drilled in the skull and a bipolar stimulating electrode and a monopolar recording electrode were twisted together and chronically implanted in the hippocampal CA1 region of the right hemisphere (AP 2.3 mm; ML1.7 mm; DV 2.6 mm below dura) (Paxinos and Watson, 1986). Apart from the cross-section of the electrodes tip, electrodes (Stainless steel, Teflon-coated, 127 μm in diameter, AM-Systems, USA) were insulated. As ground and differential electrode, an electrode was installed on skull screw, positioned above the surface of the left skull. For the head stage, all electrodes were connected to pins of a multi-channel miniature socket and fixed to the skull with dental acrylic.

One week after post-surgical recovery, after-discharge threshold was determined in hippocampus by 3 s, 50 Hz monophasic square wave stimulus of 1 ms per wave. Stimulations were initially delivered at 10 μA and then at 5 min intervals, intensity increased in increments of 10 μA until at least 5 s of After-Discharges (ADs) were recorded as previously described (Esmaeilpour et al., 2017). The animals were electrically stimulated daily at the AD threshold intensity in a semi-rapid kindling procedure (12 stimulations/day at 10 min intervals) until three consecutive stage 5 seizures (fully kindled state). The behavioral seizure severity was rated according to Racine’s scale (Racine, Rose, Burnham, 1977).

### 2.3. Passive avoidance test

The passive avoidance task is a fear-aggravated test used to evaluate associative learning and memory in rodents. The animal learns to avoid an environment in which a prior aversive stimulus has been delivered. Here, passive avoidance learning was assessed using an inhibitory passive avoidance paradigm as described here. Briefly, a shuttle-box device with dimensions of 100[L]×25[W]×25[H] (cm) consisting of two compartments (light and dark) separated by a door was used. In the learning phase of the test, each animal was first habituated to the test equipment by placing in the light chamber (door closed) for 5 minutes before returning to the home cage. The next day, the animal was returned to the light compartment, the door opened and the animal allowed moving to the dark chamber before the door was closed and the animal returned to the home cage. This process was repeated once and if an animal failed to move into the dark compartment, it was removed from the study. Finally, one hour after the previous exposure to the apparatus, the animal was placed into the light compartment, the door was opened and, on entering the dark compartment, it was given an electric shock (0.5A, 2 ms; via wires embedded in the dark chamber floor).

This final part of the process was repeated up to five times at 1 hour intervals until the animal learned to avoid the dark compartment (remains in light compartment for at least 300 s) and the number of shocks required for learning was recorded. The assessment phase of the test was undertaken 24 hours after the learning phase. The animal was placed in the light chamber (door closed) and, after 30s, the door was opened and the time took the animal to enter the dark chamber was recorded as the Step-Through Latency (STL). The total Time spent in the Dark Compartment (TDC) during a period of 5 minutes after door opening was also recorded.

### 2.4. Histology

Followed by the successive perfusion of rats by intra-cardiac injection of 0.9% saline solution and 4% Para-Formaldehyde (PFA) in 0.1 M Phosphate Buffer (PB), their brains were harvested and postfixed in 4% PFA overnight. Slices were removed after 20 minutes and after conventional paraffin embedding and serial section (5 μm), paraffin sections were made and stained with hematoxylin and eosin.

### 2.5. Experimental protocols

**Figure 1.**
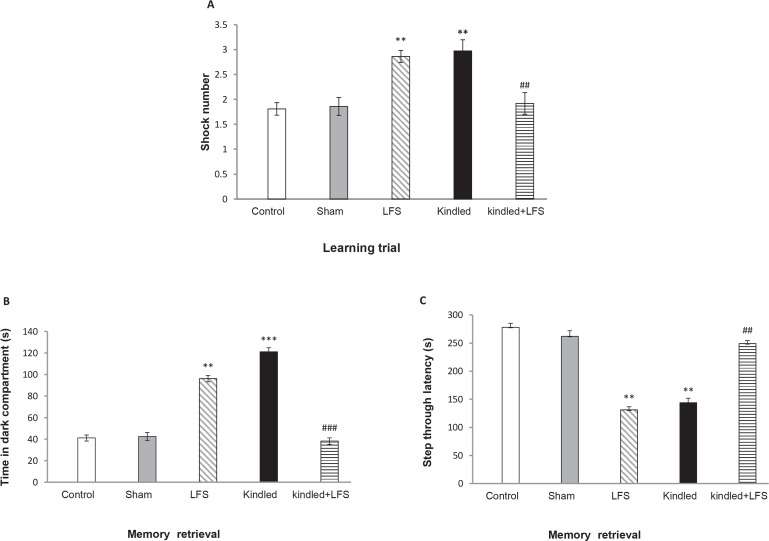
Time-line diagram showing the protocol used for LFS application in animals of kindled+LFS group

By randomly putting the animals into 5 groups of 7 rats each, we grouped them as follows: 1. Sham group which underwent operation but did not receive any kind of stimulations, 2. Kindled group consisted of fully kindled animals tested by shuttle box 48 h following the last kindling stimulation, 3) Kindled+LFS group consisted of fully kindled animals received LFS in their site of kindling stimulation (hippocampal CA1 region) so as the first and second LFS were induced immediately and then 6 h after the last kindling stimulation, third and fourth LFS were induced the next day in a similar manner (i.e. in 6 h interval). Each LFS consisted of 4 packages at 5 min intervals; each package contained 200 monophasic square wave pulses of 0.1 ms duration at 1 Hz. LFS pattern was achieved according to our initial experiments on hippocampal CA1 area. The intensity for LFS delivery was equal to AD threshold for each kindled rat. Animals were tested by shuttle box 24 h after the last LFS, 4. LFS group was manipulated similar to kindled + LFS group, however they only got LFS with no kindling stimulation, 5. Control group received shuttle box test without undergoing surgery and any kind of stimulations (Figure 1).

In all experimental groups, animals were tested by shuttle box 48 h after the last kindling stimulation in the kindled group, 24 h after the last LFS in kindled+LFS and LFS groups and at the similar time in control and sham operated animals.

### 2.6. Data analysis

Data obtained in the passive avoidance test were examined by 2-way ANOVA. When statistical significance was found between groups, Tukey’s post hoc multiple comparison test was performed to determine points of significant difference. The obtained data were averaged and presented as mean±S.E.M. P<0.05 was considered as statistically significant.

## 3. Results

**Figure 2.**
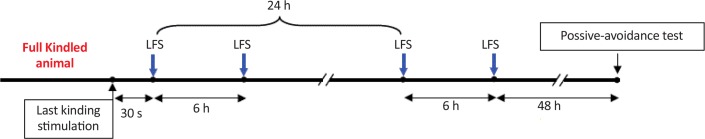
The effects of kindling and LFS on learning trial (shock number) (A) and retention test including time spent in dark compartment (B) and STL (step through latency) (C) Kindling increased shock number and time spent in dark compartment while decreased STL compared to other groups. LFS reversed this changes. Data are shown as mean±SEM. ** P<0.01 and *** P<0.001 compared to the sham group. ## P<0.01 and ### P<0.001 compared to Kindled group. In all groups n=7.

One-way ANOVA results indicated that 48 h after the last kindling stimulation, the number of shock received (Figure 2A) in animals of kindled group was significantly higher than those of sham group during the training session (P<0.01). This indicates learning impairment in kindled animals. Application of 4 packages of LFS in kindled+LFS group decreased the number of shocks in comparison to kindled group (P<0.01) and improved kindling-induced impairment in associative learning 48 h after the last kindling stimulation. No significant difference was seen between kindled+LFS and sham group (Figure 2A). Despite the refining action of LFS in kindled + LFS group, administration of LFS alone increased the shock number (Figure 2A) compared to the sham group in training session. It means that the response of LFS group was similar to that of the kindled group.

The test session was performed 24 h following the training session and the Step Through Latency (STL) and Time in Dark Compartment (TDC) were analyzed to assess memory retrieval. The Time in the Dark Compartment (TDC) was also altered in the kindled group in comparison with the sham groups (P<0.01). LFS counteracted this effect of kindling on TDC in kindled+LFS group (P<0.001) (Figure 2B). Tukey’s test following 1-way ANOVA showed that in animals of kindled group, STL decreased (P<0.01) compared with the sham group (Figure 2C). This clearly indicates memory impairment in these animals. Application of LFS in kindled+LFS group ameliorated memory deficit. In kindled+LFS group, STL increased (P<0.01) compared to kindled group. In addition, no significant difference was observed between Kindled+LFS and sham group. Similar to kindled group, application of LFS alone resulted in memory impairment (Figure 2C). In addition, Tukey’s post hoc test did not indicate any significant difference between the control and sham experimental groups.

**Figure 3.**
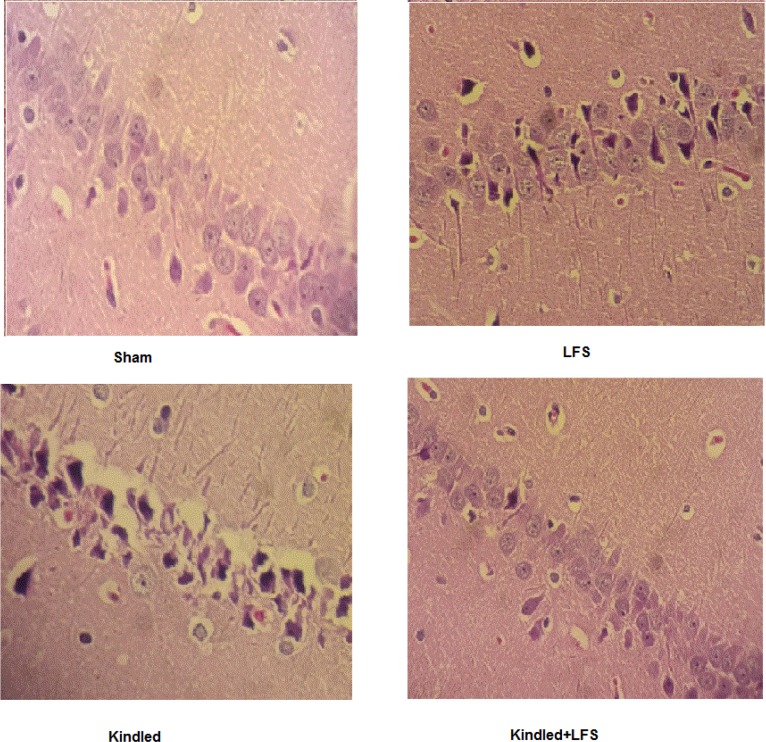
Hematoxylin and Eosin (H&E) staining Both hippocampal atrophy and significant neuronal loss in CA1 was observed in kindled group. Treatment with LFS reduced hippocampal atrophy and hippocampal CA1 area neuronal loss in kindled+LFS.

As expected, results of histology and behavioral studies were consistent with behavioral experiments. Both hippocampal atrophy and significant neuronal loss in CA1 was observed in kindled group. Treatment with LFS reduced hippocampal atrophy and hippocampal CA1 area neuronal loss in kindled+LFS (Figure 3).

## 4. Discussion

Our findings indicate that hippocampal kindling leads to impairment of learning in the passive avoidance test. However, application of LFS in CA1 region of the dorsal hippocampus can prevent the kindled seizure-induced impairment of emotional learning in fully kindled animals.

When memory was assessed in the passive avoidance test, kindling-impaired acquisition of passive avoidance is demonstrated by a shorter step through latency and longer time spent in the dark compartment. Application of LFS modulates this kindling-induced impairment since LFS induction partially reversed the kindling-induced reduction in step through latency and increase in time spent in the dark compartment.

These findings were in accordance with the earlier studies suggesting that memory deficit is associated with the kindling process. Previous studies have shown that hippocampal kindling impairs spatial memory deficits in the radial arm maze (Leung et al., 1990; Stan Leung & Shen, 1991), Morris water maze (Gilbert et al., 1996), and passive avoidance test (Grecksch et al., 1991; Pohle et al., 1997; Hannesson et al., 2001; Szyndler et al., 2002). LTP phenomenon has a crucial role in learning and memory in hippocampus, so that any changes in hippocampal LTP may be caused by kindling-induced learning impairment (Schubert, Siegmund, Pape, & Albrecht, 2005; Mazarati, 2007). Kindling induces synaptic potentiation which is similar to LTP (Cain 1989; Cain, Boon, Hargreaves, 1992). It is believed that this potentiation prevents of producing a new plasticity in hippocampal synapses. This saturation in synaptic plasticity can disrupt learning and memory in kindled animals (Schubert et al., 2005).

LFS induces Long-Term Depression (LTD) (Fujii et al., 2000; Manahan-Vaughan, 2003) and depotentiation (Manahan-Vaughan, 2003; Klausnitzer, Kulla, Manahan-Vaughan, 2004). Therefore, it is reasonable to conclude that the suppressing effect of LFS on kindling-induced synaptic potentiation is one of the best reasons for improving effect of LFS on shuttle box performance of fully kindled rats. Our previous experiments also show that application of LFS prevents kindling-induced synaptic potentiation in perforant path-granular cells synapses (Mohammad-Zadeh et al., 2007; Jahanshahi, Mirnajafi - Zadeh, Javan, Mohammad-Zadeh, & Rohani, 2009).

The present finding also supports our previous research which concluded that LFS can improve spatial learning and memory impairments induced by hippocampal kindling in both MWM (Esmaeilpour et al., 2017) and Y-maze task (Ghafouri et al., 2016).

Histological assessment shows that administration of LFS following kindling stimulations could prevent the cell loss in hippocampal area of the fully kindled animals. Hippocampal cell loss may contribute to the impairment of learning following seizure induction (Pauli, Hildebrandt, Romstock, Stefan, & Blumcke, 2006). Therefore, the improving effect of LFS in our study may partly be because of its protective effect against seizure-induced neuronal degradation.

Interestingly, while LFS improves the situation of kindled animals, its application alone in normal rats has destructive effects on emotional learning (similar to kindling stimulations). This difference in the effectiveness of LFS might be related to the neural activity level. On the other hand, the history of neuronal activity is a very important factor in determining its effectiveness.

In conclusion, the findings of this study demonstrated that LFS attenuates kindling-induced impairments of emotional learning and memory in the kindled rats. The results suggest that LFS can be used as a potentially novel therapeutic method in patients with epilepsy.
